# HO-1 promotes resistance to an EZH2 inhibitor through the pRB-E2F pathway: correlation with the progression of myelodysplastic syndrome into acute myeloid leukemia

**DOI:** 10.1186/s12967-019-2115-9

**Published:** 2019-11-11

**Authors:** Zhengchang He, Siyu Zhang, Dan Ma, Qin Fang, Liping Yang, Shaoxian Shen, Ying Chen, Lingli Ren, Jishi Wang

**Affiliations:** 1 Department of Hematology, Hematological Institute of Guizhou Province, Guizhou Provincial Laboratory of Hematopoietic Stem Cell Transplantation Centre, Affiliated Hospital of Guizhou Medical University, Guiyang, 550004 Guizhou People’s Republic of China; 2 Department of Pharmacy, The Affiliated Hospital of Guizhou Medical University, Guiyang, People’s Republic of China; 3 Clinical Medicine Research Center of Affiliated Hospital of Guizhou Medical University, Guiyang, Guizhou People’s Republic of China; 4 Intensive Care Unit, Affiliated Hospital of North Sichuan Medical College, Nanchong, Sichuan People’s Republic of China; 5 Department of Hematology, Affiliated Hospital of North Sichuan Medical College, Nanchong, Sichuan People’s Republic of China

**Keywords:** Myelodysplastic syndrome, Acute myeloid leukemia, pRB-E2F, JQEZ5, HO-1, EZH2

## Abstract

**Background:**

Myelodysplastic syndrome (MDS) can progress to acute myeloid leukemia (AML), and conventional chemotherapy (decitabine) does not effectively inhibit tumor cells. Enhancer of zeste homologue 2 (EZH2) and Heme oxygenase-1 (HO-1) are two key factors in patients resistance and deterioration.

**Methods:**

In total, 58 MDS patients were divided into four groups. We analyzed the difference in HO-1 and EZH2 expression among the groups by real-time PCR. After treatment with Hemin or Znpp IX, flow cytometry was used to detect apoptosis and assess the cell cycle distribution of tumor cells. Following injection of mice with very high-risk MDS cells, spleen and bone marrow samples were studied by immunohistochemistry (IHC) and hematoxylin and eosin (H&E) staining. MDS cells overexpressing EZH2 and HO-1 were analyzed by high-throughput sequencing. The effect of HO-1 on the pRB-E2F pathway was analyzed by Western blotting. The effects of decitabine on P15INK4B and TP53 in MDS cells after inhibiting HO-1 were detected by Western blotting.

**Results:**

Real-time PCR results showed that EZH2 and HO-1 expression levels were higher in MDS patients than in normal donors. The levels of HO-1 and EZH2 were simultaneously increased in the high-risk and very high-risk groups. Linear correlation analysis and laser scanning confocal microscopy results indicated that EZH2 was related to HO-1. MDS cells that highly expressed EZH2 and HO-1 infiltrated the tissues of experimental mice. IHC results indicated that these phenomena were related to the pRB-E2F pathway. High-throughput sequencing indicated that the progression of MDS to AML was related to EZH2. Using the E2F inhibitor HLM006474 and the EZH2 inhibitor JQEZ5, we showed that HO-1 could regulate EZH2 expression. HO-1 could stimulate the transcription and activation of EZH2 through the pRB-E2F pathway in MDS patients during chemotherapy, which reduced TP53 and P15INK4B expression.

**Conclusions:**

EZH2 was associated with HO-1 in high-risk and very high-risk MDS patients. HO-1 could influence MDS resistance and progression to AML.

## Background

Myelodysplastic syndrome (MDS) is a clonal hematopoietic stem cell disorder that can progress to acute myeloid leukemia (AML) [1]. The pathogenesis of MDS is controversial, with epigenetic regulation being one of the main pathogenic mechanisms [2]. We grouped patients according to the World Health Organization (WHO) prognostic scoring system (WPSS). Currently, some high-risk and very high-risk MDS patients are resistant to decitabine and therefore have a poor prognosis [3]. A study indicates that the median survival time of these patients is less than 0.8 years, which was significantly less than the median survival time of low-risk patients (5.3 years). 25% of high-risk patients convert to AML within 1.4 years [4]. We noticed that enhancer of zeste homologue 2 (EZH2) could affect the self-renewal and differentiation of hematopoietic stem cells [5] and may be an independent prognostic factor [6] related to a poor prognosis [7]. EZH2 expression is also abnormally upregulated in AML. A study indicated that combining inhibition of EZH2 and LSD1 resulted in synergistic activity against AML in vitro and in vivo. This synergy was mechanistically correlated with upregulation of H3K4me1/2 and H3K9Ac and downregulation of H3K27me3, leading to a decrease in the level of the antiapoptotic protein Bcl-2 [8]. Due to the role of EZH2 in the treatment of AML, we speculated that this gene may contribute to MDS progression to AML and the rise of drug-resistant AML and MDS phenotypes. Other studies have also shown that EZH2 can increase cancer cell aggressiveness [9–11]. It is not known whether MDS patients can easily progress to AML following EZH2 overexpression. It is also unclear whether EZH2 inhibition has inhibitory effects on high-risk or very high-risk MDS progression.

Heme oxygenase-1 (HO-1) is an antioxidant enzyme involved in many cellular processes, including oxidative stress and apoptosis regulation. Several studies have indicated that HO-1 can affect leukemic cells [12, 13], but the mechanisms involved require further investigation [14]. HO-1 has antiapoptotic effects on MDS cells [15, 16]. It is worth noting that EZH2 and HO-1 share similar functions [17]. For example, overexpression of HO-1 or EZH2 can significantly inhibit patients’ response to decitabine which is related to patients’ prognosis. To date, the effects of the HO-1 gene on inhibitors of EZH2 have never been reported. In recent years, the development of MDS therapies has been slow, and we hope to provide a new perspective. In this study, we mainly explored MDS patient models that were different from the models in previous studies.

## Materials and methods

### Ethical declaration

Our research was approved by the Committee on Animal Protection and use of the Animal Management Board of the United States. Conducting relevant animal experiments according to approved guidelines, we follow all applicable international, national and institutional guidelines for animal care and use. The experiment was strictly followed the Helsinki declaration and passed the ethical examination of animal experiments in Guizhou Medical University. (Ethical approval number: 2017-13)

### Cells and culture conditions

At present, many famous biotech companies have not cultivated MDS cell lines recognized by blood experts. MDS cells are also unstable. So we choose to extract patient cells. All human MDS cells and AML cells were preserved in our laboratory. We collected MDS cells and MDS cells that progress to AML. All cells are clearly grouped and divided into treated and untreated. All cells were cultured with 15% fetal bovine serum (Gibco BRL; Life Technologies, Carlsbad, CA, USA), penicillin of 100  μ/ml and RPMI-1640 culture of streptomycin in 100 μg/ml. All cells were kept at 37 °C incubator with humidity of 95% and CO_2_ content of 5%. RPMI-1640 medium was purchased from Invitrogen (Carlsbad, CA, USA).

### Patient samples

Fifty-eight patients who were diagnosed as MDS and received related treatment all the time (Table 1). According to the Helsinki declaration, the informed consent was firstly obtained in writing, and then samples of bone marrow blood or peripheral blood are extracted. All patients were classified according to the WHO prognostic scoring system. The bone marrow blood and peripheral venous blood of the patients or normal donors were extracted by a proper amount of lymphocyte separation fluid Ficoll (Sigma Chemical Co.). All extracted cells were incubated at the same time and then tested. And remove all external interference factors. We ensure that all extracted cells are representative of the biological characteristics of the patients. The study was approved by the institutional review board (Affiliated Hospital of the Guiyang Medical College).

**Table 1:** Patient characteristics

Patient characteristics	
*Characteristic*	*(n,* %*)*
Age (years)	58
<70	40 (69%)
≥70	18 (21%)
Sex	58
Male	44 (76%)
Female	14 (24%)
WHO classification	58
RA/RAS	10 (17%)
RCMD/RSCMD	14 (24%)
RAEB-I	9 (16%)
RAEB-II	22 (38%)
RAEB-t/AML	3 (5%)
WPSS risk group	58
Very low	6 (10%)
Low	9 (16%)
Intermediate	14 (24%)
High	13 (22%)
Very high	16 (28%)

### Virus transfection

Self-prepared sequences containing human HO-1 and small interfering RNA targeting human HO-1 were selected with Invitrogen designer software. Retroviruses were generated by transfecting empty plasmid vectors containing the enhanced green fluorescence protein (EGFP) or vectors containing human HO-1-EGFP/siRNA-HO-1-EGFP into 293FT packaging cells, using the FuGENE HD6. Lentiviral stocks were concentrated using Lenti-X concentrator, and titers were determined with Lenti-X qRT-PCR titration kit (Shanghai Innovation Biotechnology Co., Ltd., China). Finally, 4 recombinant lentiviral vectors were constructed: lentivirus-V5-D-TOPO-HO-1-EGFP (L-HO-1), lentivirus-V5-D-TOPO-EGFP (TOPO-EGFP), lentivirus-pRNAi-u6.2-EGFP-siHO-1 (siHO-1), and lentivirus-pRnai-u6.2-egfP (RNAi-EGFP). For transduction, cells were plated onto 12-well plates at the density of 2.5 × 105/well, infected with the lentiviral stocks at a multiplicity of infection of 10 in the presence of polybrene (10 µg/ml), and then analyzed by fluorescence microscopy (Olympus, Tokyo, Japan) and Western blotting 48 h after transduction. MDS cells were transduced with L-HO-1, siHO-1, RNAi-EGFP and TOPO-EGFP, respectively. Titer: L-HO-1: 4.0 × 10^9^TU/ml, si-HO-1: 8.0 × 10^8^TU/ml, EV1-siHO-1: 6.0 × 10^8^TU/ml, EV2-L-HO-1: 4.0 × 10^8^TU/ml.

### RNA isolation and quantitative PCR

Total RNAs from cells were extracted using Trizol reagent (Invitrogen, Carlsbad, CA, USA). Quantitative PCR was performed by using SYBR Green PCR Master Mix (TianGen, Biotech, Beijing, China) and the PRISM 7500 real-time PCR detection system (ABI, USA). cDNA samples, primers and SYBR Master Mix were mixed with a total volume of 20ul. The thermal cycling conditions used in the protocol were 1 min at 94 °C, followed by 40 cycles at 94 °C for 10 s and at 60 °C for 15 s.

### Reagents

HO-1 enhancer Hemin was purchased from Sigma (St Louis, MO, USA). HO-1 inhibitor Znpp IX was purchased from Cayman Chemical (Ann Arbor, MI, USA). EZH2 inhibitor JQEZ5 and E2F inhibitor HLM006474 were purchased from MCE (Shanghai, China). The drugs were dissolved in a small amount of DMSO and stored in − 20 °C. Before using, we used serum-free RPMI-1640 to dilute it. Annexin V-fluorescein isothiocyanate (FITC)/propidium iodide (PI) apoptosis detection Kit was purchased from BD (San Diego, CA, USA). qPCR primers such as HO-1 and EZH2 were synthesized by Sangon Biotech (Shanghai, China). Western-blot was probed with primary antibodies, including antibodies against HO-1, EZH2, pRB. Secondary antibodies were purchased from Li-Cor Corp (Lincoln, Nebraska, USA). The TRIzol of total RNA extracted from cells and mononuclear cells was purchased from Invitrogen (Carlsbad, CA, USA).

### Apoptosis analysis

After PI staining, cell apoptosis was detected by flow cytometer (BD Biosciences, San Jose, CA, USA). The cells were washed with phosphate buffer saline (PBS), resuspended in 100 μl of binding buffer containing 5 μl of annexin V and finally stained with 5 μl of PI at room temperature in dark for 15 min.

### Cell cycle analysis

Cell cycles were detected by flow cytometry. The cells were washed with PBS, fixed in 70% cool ethanol for 2 h, washed with PBS again, and resuspended in PBS containing PI (Sigma), DNase free RNase (Citomed) and Triton X-100 for 1 h. Finally, the cells were collected by FACS Calibur flow cytometer (Becton-Dickinson), and cell cycle-related data were analyzed by FlowJo software (Tree Star, Inc., Ashland, OR, USA).

### Western blot analysis

Western blot was employed to detect the protein expressions of related genes in patients with different MDS risks, as well as in the cells transfected by siRNA or lentivirus in combination with JQEZ5 and decitabine treatment. The expression of proteins in blood samples or treated cells were analyzed by Western-blot. PBS were lysed by sonication in RIPA buffer (the cells were lysed sonication in RIPA buffer). The cells were fully mixed and transferred to the new EP tube, and then were centrifuged at 12,000*g for 10 min at 4 °C. After centrifugation, the supernatant was mixed with loading buffer and stored at − 80 °C. After loading the same amount of protein (50–100 μg) with 10% SDS-PAGE, electrophoresis was separated and then was transferred to the PVDF membrane (Millipore Corporation, Milford, MA, USA). The protein PVDF was transferred to the TRIS buffer which contained 5% skim milk powder overnight. The membrane was blotted with relevant primary antibodies (1:1500) for 2 h. After being washed with PBS and 0.1% Tween-20, the blot was incubated with secondary antibody (1:2000). The expression level of related proteins was determined by enhanced chemiluminescence (7sea Biotech, Shanghai, China). Each experiments was conducted more than 3 times.

### Animals and treatments

Male C57BL/6Ly5.2 mice weighing 20–21 g were purchased from the Institute of Laboratory Animal Sciences (PUMC, Beijing, China). Mice were cultured in SPF class (SPF, Specific Pathogen Free) animal laboratory. After being adapted to the environment, the 10 mice were divided into two groups randomly. One group of five mice were served as control group and were only injected culture medium. The remaining groups of mice were experimental group. (each mice was injected 3 × 10^7^ U266 cells). All mice were injected via tail vein every 2 days for 4 weeks. The loss of weight and survival time of mice were recorded and analyzed. immunohistochemistry (IHC) and hematoxylin and eosin (HE) staining were used to detect MM cell infiltration in liver, spleen, kidney. All experiments were conducted at least three times.

### Statistical analysis

Each experiment was repeated at least 3 times and the most representative example was given. Statistical analysis of experimental data was performed by using GraphPad Prism 5 software (GraphPad Software Inc, San Diego, CA, USA). All data were represented as mean ± standard error. Statistical analyses were performed by using analysis of variance and the *t* test. Results were considered statistically significant if P < 0.05 and data were represented as mean ± standard deviation (SD) of three independent experiments (*P < 0.05; **P < 0.01; ***P < 0.001).

## Results

### EZH2 and HO-1 are relevant in some high-risk and very high-risk MDS patients

**Fig. 1:**
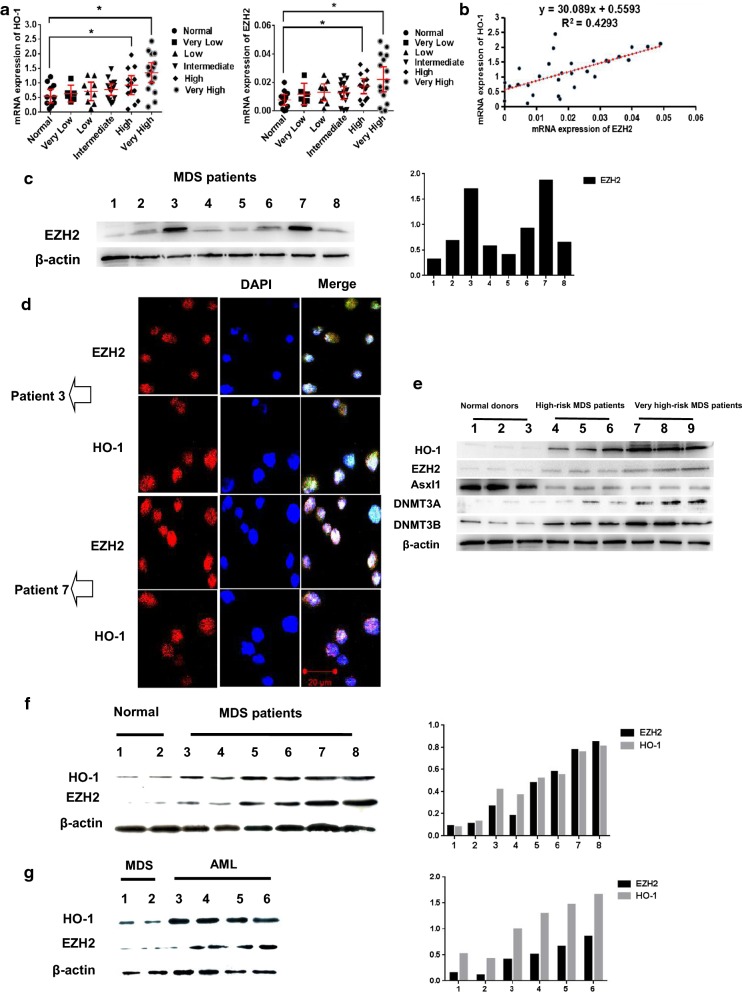
EZH2 and HO-1 are relevant in some high-risk and very high-risk MDS patients. **a** Mononuclear cells were extracted from the bone marrow blood of newly diagnosed patients. cDNA and protein were extracted, and EZH2 and HO-1 were detected by real-time PCR. The basic information of the MDS patients is shown in Table 1. **b** Linear correlation analysis was used to evaluate EZH2 and HO-1 mRNA expression in high-risk and very high-risk MDS patients. R^2^ represents the correlation between the two genes. **c** In total, 8 MDS patients with the highest HO-1 expression in qPCR results were selected, and the results were validated by Western blotting to detect EZH2 expression. We also used the Quantity one software to analyze gray values. The relevant values are shown in the histogram. **d** The levels of proteins in the cells were detected by the laser scanning confocal microscopy. DAPI was ued for nuclear staining. Merge refers to the overlap image. **e** Western blotting was used to detect the correlation genes expression between EZH2 and HO-1 gene expression in high-risk and very high-risk MDS patients and normal donors. **f** Western blotting was used to detect EZH2 and HO-1 expression in MDS patients and normal donors. We also used Quantity one software to analyze gray values. The relevant values are shown in the histogram. **g** Western blotting was used to detect EZH2 and HO-1 expression in different groups of MDS patients. The patients were divided into two groups. One group comprised MDS patients with a good prognosis. The other group comprised AML patients who progressed from MDS and were not sensitive to chemotherapy (decitabine). We also used Quantity one software to analyze gray values. The relevant values are shown in the histogram. Scale bars, 20 μm. Western blotting bands were quantified with Quantity One software. Each sample was normalized to related β-actin expression. All the experiments were repeated, *P < 0.05 (statistically significant)

According to the WPSS, we divided 58 MDS patients into four different groups. Bone marrow blood was extracted, and mononuclear cells were collected. Real-time PCR results showed that the expression of EZH2 and HO-1 in some MDS patients was higher than that in normal donors. HO-1 and EZH2 expression levels were simultaneously increased in some patients, especially those in the high-risk and very high-risk groups (Fig. 1a). In addition, the expression of these moleculs correlated in the high-risk and very high-risk groups (R^2^ = 0.429) (Fig. 1b). We selected 8 MDS patients who progressed to AML and showed by Western blotting elevated EZH2 expression in two patients (patient 3 and patient 7) (Table 2). We also used a bar graph to show the gray values (Fig. 1c). Western blotting results indicated that EZH2 expression differed within the MDS-risk patient group. We used laser scanning confocal microscopy to examine previous samples from patients 3 and 7 (Fig. 1d). We analyzed gene expression in MDS patients and normal donors. We found that HO-1, EZH2, DNMT3A, and DNMT3B were highly expressed in high-risk and very high-risk MDS patients. Asxl1 was highly expressed in the normal donors (Fig. 1e). We also found a correlation between EZH2 and HO-1 expression by Western blotting. The associated bar graph shows the gray values (Fig. 1f). When we selected four MDS samples from patients who progressed to AML, we found that EZH2 and HO-1 expression was significantly higher in the progressed MDS patients than in other MDS patients. We also used a bar graph to show the gray values (Fig. 1g). Taken together, the data indicate that HO-1 and EZH2 expression may be relevant in high-risk and very high-risk MDS patients.

**Table 2:** Patient characteristics

Patient	Age (years)	Sex	WPSS risk group	Blood cells	Primitive cells (%)	Auer rod	No. of monocytes
Patient 3	61	Male	High	Decrease	18	Positive	0.8 × 10^9^/L
Patient 7	65	Male	Very high	Decrease	13	Positive	0.7 × 10^9^/L
Patient 1	63	Male	Very high	Decrease	15	Positive	0.4×10^9^/L

### JQEZ5 significantly promotes MDS cell apoptosis

**Fig. 2:**
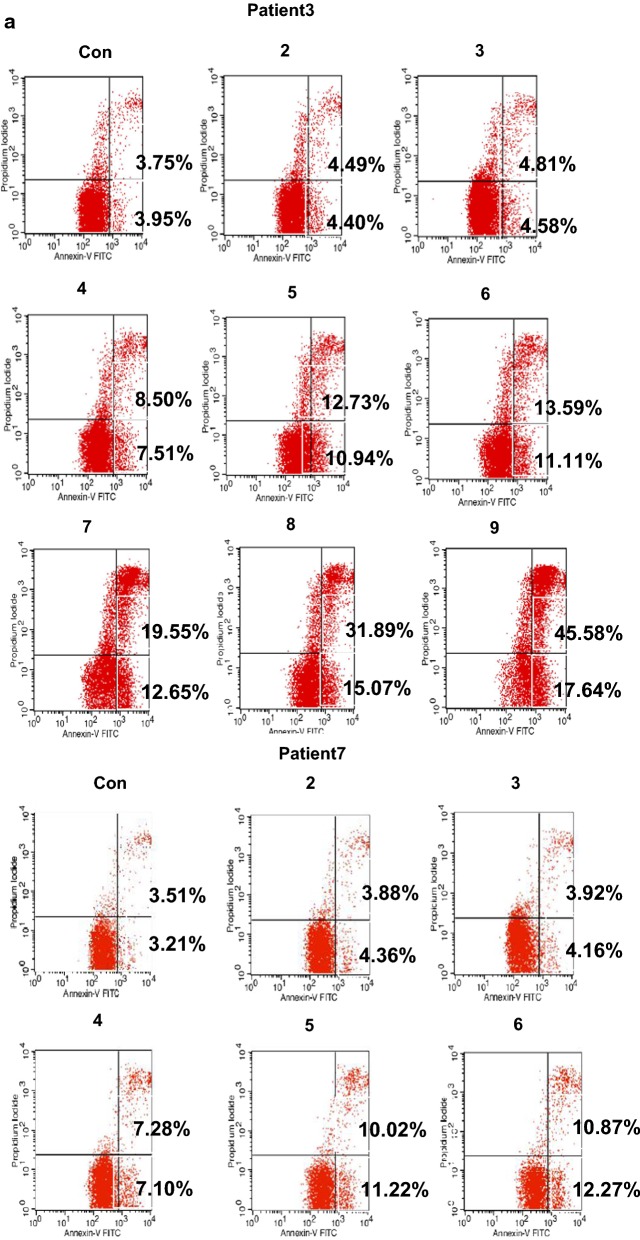

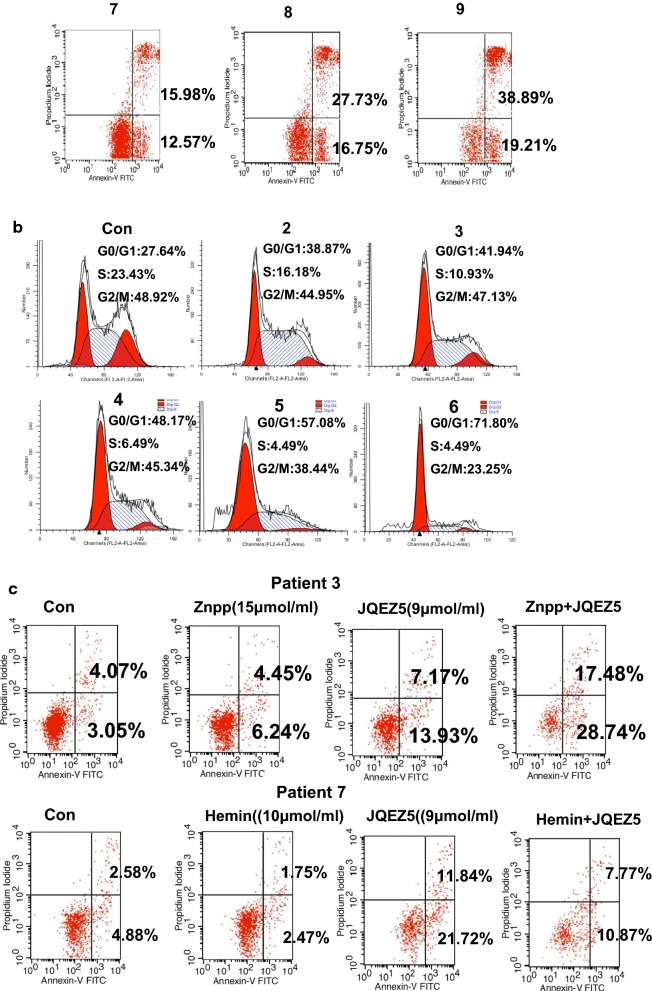

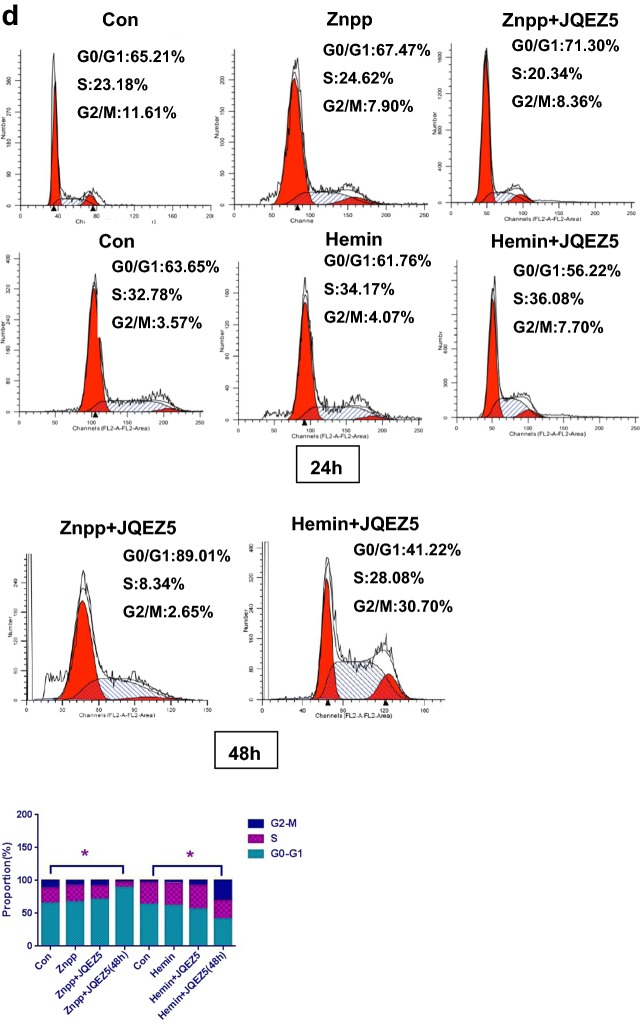
JQEZ5 significantly promotes MDS cell apoptosis. **a**The basic information of MDS patients (patient 3 and patient 7) is shown in Table 2. We selected two typical MDS patients (patient 3 and patient 7) for subsequent experiments. They were both determined to have high expression of EZH2 and HO-1 in previous experiments. The control groups (Con) were not treated with JQEZ5. We prepared different concentrations of JQEZ5, and then cultured cells isolated from the two patients with different concentrations of JQEZ5 for 24 h. We used flow cytometry to detect apoptosis rates and perform comparisons. The bar graph indicates the percentage of annexin V-positive cells (apoptotic cells). **b** We used flow cytometry to detect the cell cycle distribution of the above experimental groups. The bar graph shows the percentages of cells in different phases of the cell cycle. **c** HO-1 expression in patient 3 cells was downregulated by Znpp, and HO-1 expression in patient 7 cells was upregulated by Hemin. The patient 7 cells were divided into Con (control), Znpp (treated with 15 μmol/ml ZnppIX), JQEZ5 (treated with 9 μmol/ml JQEZ5) and Znpp + JQEZ5 (treated with 15 μmol/ml ZnppIX and 9 μmol/ml JQEZ5) groups. The patient 3 cells were divided into Con (control), Hemin (treated with 10 μmol/ml Hemin), JQEZ5 (treated with 9 μmol/ml JQEZ5) and Hemin + JQEZ5 (treated with 10 μmol/ml ZnppIX and 9 μmol/ml JQEZ5) groups. **d** The cell cycle distribution of the above experimental groups were determined. The cell cycle distribution of each group was analyzed by flow cytometry. The Con groups were not treated with any drug. All the experiments were repeated, *P < 0.05 (statistically significant)

We selected two previously evaluated MDS patients (patient 3 and patient 7) because they were not sensitive to chemotherapy and rapidly progressed to AML. To clarify whether an EZH2 inhibitor can affect MDS cells, we used JQEZ5 to treat cells isolated from patient 3 or patient 7. Cells in the control groups (Con) were not treated with JQEZ5. We experimented with different concentrations of JQEZ5 (5 μmol/ml, 6 μmol/ml, 7 μmol/ml, 8 μmol/ml, 9 μmol/ml, 10 μmol/ml, 12.5 μmol/ml, 15 μmol/ml, and 20 μmol/ml groups). The apoptosis rate was related to the JQEZ5 concentration. As the JQEZ5 concentration increased, more apoptosis was observed in MDS cells. We also used a bar graph to show the trend in each apoptosis rate (Fig. 2a). The proportion of MDS cells in the G0/G1 phase increased when the cells were treated with JQEZ5. These results indicated that JQEZ5 mainly caused tumor cells to arrest in the G0/G1 phase of the cell cycle. We used a bar graph to represent the cell cycle (Fig. 2b) and Hemin and Znpp to regulate HO-1 expression. We found that Hemin could attenuate the proapoptotic effect of JQEZ5, whereas Znpp had the opposite effect. We used two bar graphs to show the apoptosis rates **(**Fig. 2c). The Con groups were not treated with any drug. More cells were arrested in the G0/G1 phase when Znpp and JQEZ5 were used at the same time than when these drugs were wsed as monotherapies. In contrast, fewer cells were arrested in the G0/G1 phase when Hemin and JQEZ5 were used at the same time (48 h). We also used a bar graph to show the trend (Fig. 2d).

### MDS cells overexpressing EZH2 and HO-1 inhibit the expression of P15INK4B and TP53 in mice

**Fig. 3:**
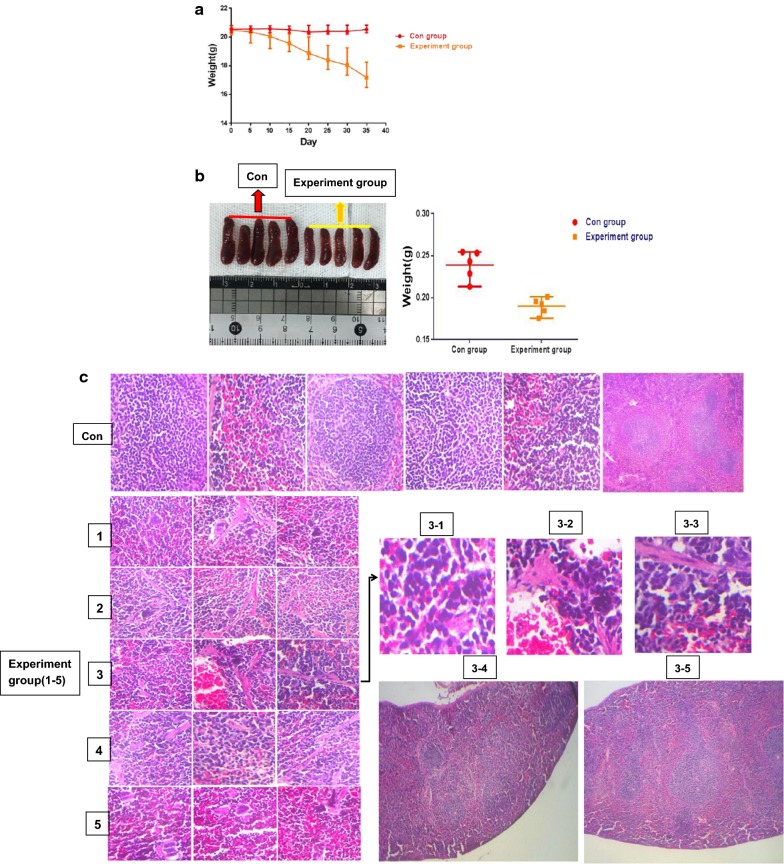

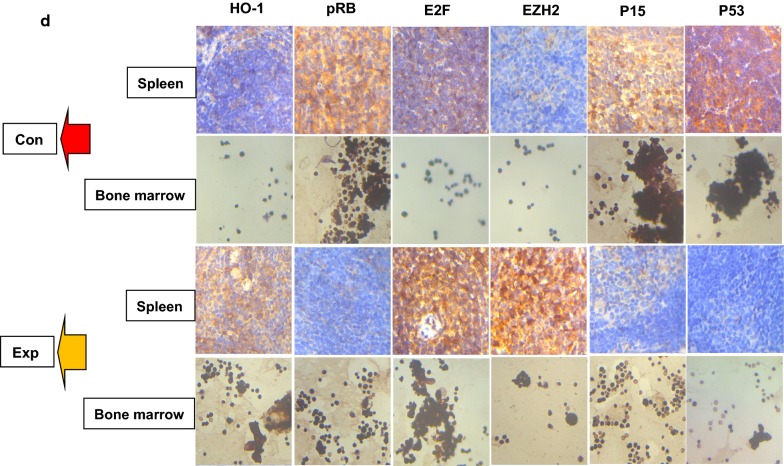
MDS cells overexpressing EZH2 and HO-1 inhibit the expression of P15INK4B and TP53 in mice. **a** MDS mouse models were established with male C57BL/6Ly5.2 mice. Five mice were included in the control group and injected with only compete medium containing 15% fetal bovine serum for tumor cell culture. The other 5 mice were included in the experimental group. Tumor cells were injected via the tail vein. In the experimental group, per mice were injected with 3 × 10^7^ MDS cell suspension. We injected the mice every day and measured body weight every 5 days. The weights of the mice were recorded. **b** We harvested the spleens from the control and experimental mice. Then we recorded spleen size and weight. **c** The spleen sections were evaluate by hematoxylin and eosin (H&E) staining. The infiltration of the tumor cells into various organs were observed. Magnification, ×400 or ×800. **d** Immunohistochemical (IHC) staining was used to evaluate organ sections. The images show positive reactions for HO-1, EZH2, pRB, E2F, P15INK4B (P15) and TP53 (P53). Magnification, ×400. Positive staining intensity was scored as 1. Cells that were not stained were scored as 0. Light brown cells that were weakly positive were scored as 1. Cells that were brown with no background staining or cells that were dark brown, but with a light brown background, (medium positive) were scored as 2. Cells that were dark brown with no background staining (strongly positive) were scored as 3. We showed the most representative images. All the experiments were repeated, *P < 0.05 (statistically significant)

We knew that patient 3 quickly progressed to AML, was insensitive to chemotherapy and unfortunately died quickly. We hypothesized that this pattern was related to EZH2 and HO-1. To investigate this hypothesis, we set up in vivo experimental (EXP) and control (Con) groups, including 5 mice (20–21 g) per group. The control mice were injected with culture medium every 2 days, and the experimental mice were injected with MDS patient cells (patient 3) every 2 days. All mice were injected via the tail vein and housed in an specific pathogen-free (SPF) animal laboratory for 40 days. The weights of the mice were measured every 5 days. Compared with the control mice, the mice injected with MDS cells lost weight, with one mouse losing 4.1 g (Fig. 3a). We also compared spleen size among all mice and found that the spleens of the mice in the experimental group were smaller and their weights were lighter (Fig. 3b). We stained spleen sections by hematoxylin and eosin (H&E) staining, and we investigated the structures of the white and red pulps that could clearly be seen in the control spleen sections. Notably, the red pulp regions of the experimental group were significantly enlarged (3–4 and 3–5). In addition, tumor-induced myeloid hyperplasia and abnormal megakaryocyte proliferating were observed in the red pulp of the experimental group. We also observed immature myeloid cells (1–5). These cells had irregular nuclei and chromatin edge set (3–1, 3–2 and 3–3) (Fig. 3c). In addition, we stained the spleen and bone marrow by immunohistochemistry (IHC) and compared the results of the experimental and control groups. IHC showed that HO-1, EZH2 and E2F were positively stained in the bone marrow and spleen of the experimental mice. It also showed that pRB, P15INK4B (P15) and TP53 (P53) were positively stained in the bone marrow and spleen of the control mice (Fig. 3d). Therefore, we hypothesized that the pRB-E2F pathway might induce the activation of EZH2 in MDS cells.

### Characteristics of MDS cells overexpressing EZH2 and HO-1

**Fig. 4:**
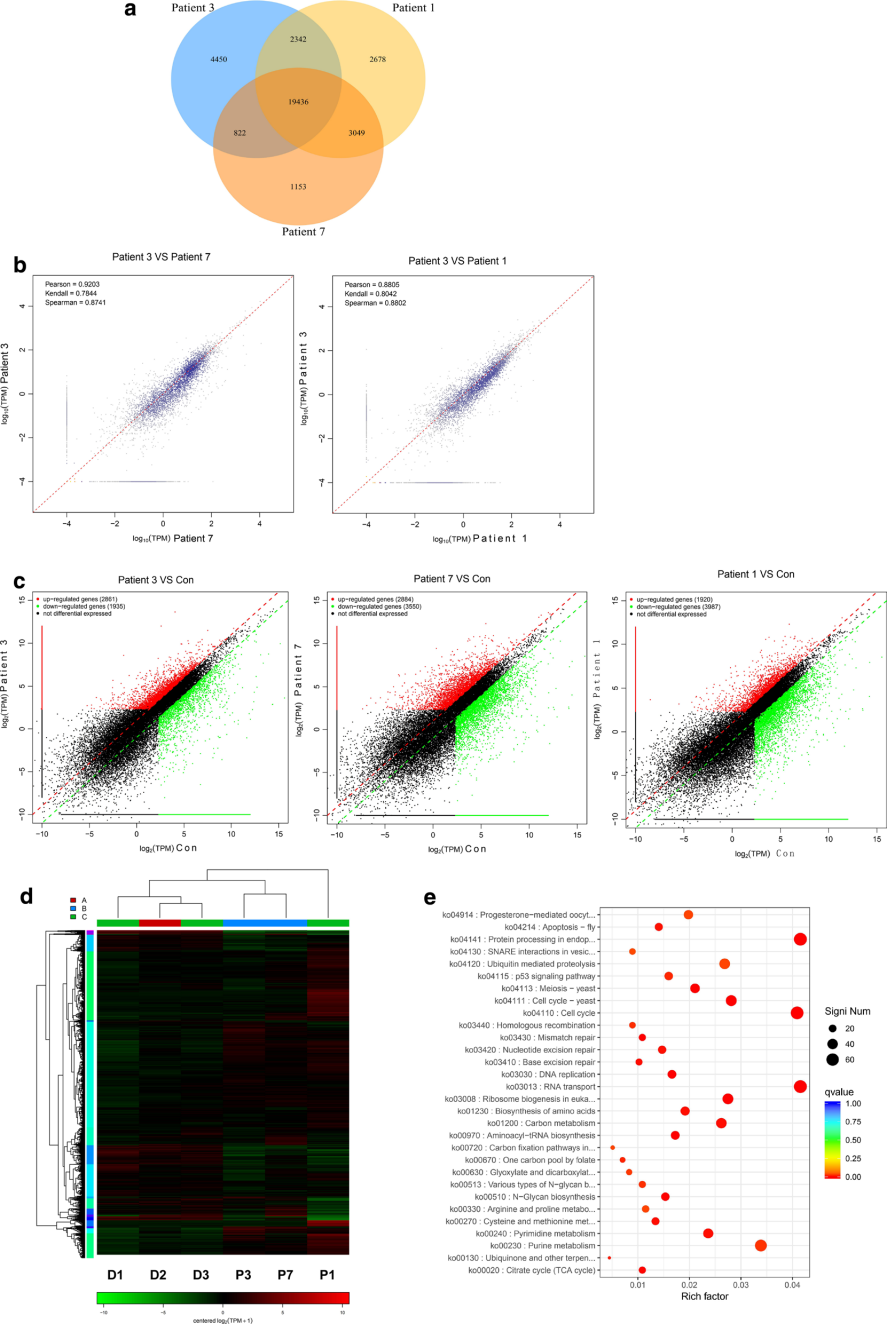
Characteristics of MDS cells overexpressing EZH2 and HO-1. **a** The basic information of MDS patients (patient 1, patient 3 and patient 7) is shown in Table 2. These three MDS patients progressed to AML in the later stages. They had experienced both the MDS phase and AML phase. We collected cells from these two phases and cultured them. To ensure the accuracy of the results, we did not culture the cells for long periods. Gene expression was analyzed by high-throughput techniques. The overlapping parts represent the same genes. **b** The **s**catterplot shows a correlation among the patients. The Pearson correlation coefficient was used to measure whether two data sets were above a line. It was used to measure the linear relationship between distance variables. A correlation coefficient of 0.8–1.0 represents an extremely strong correlation. **c** Gene expression differences between three samples and normal donors were compared. The red dots represent the upregulated genes. The green dots represent the downregulated genes. The black dots represent no gene with no difference in genes expression. **d** Three patients were analyzed by gene chip analysis. Because they all overexpressed EZH2 and HO-1, there were many similarities. D1, D2 and D3 are 3 normal donors. P1 indicates patient 1. P3 indicates patient 3. P7 indicates patient 7. **e** The genes identified in three samples were analyzed by Kyoto Encyclopedia of Genes and Genomes (KEGG) enrichment. All the experiments were repeated, *P < 0.05 (statistical significant)

Some MDS patients progressed to AML in later stages, and their isolated cells, which were injected into mice, overexpressed EZH2 and HO-1 and were able to infiltrate the spleen and bone marrow of mice. To further understand the characteristics of these MDS cells, we analyzed their genes by high-throughput sequencing. Although the three studied patients progressed to AML, the samples we selected were different. Patient 1 had progressed to AML. Patient 3 and patient 7 had not progressed to AML (Table 2). We found that samples from these three patients had many abnormally expressed genes (Fig. 4a). The gene expression of patient 3 and patient 7 correlated. The expression in patient 3 and patient 1 also correlated (Fig. 4b). However, when comparing the data from three patients with normal donor data (Fig. 4c), the results of gene chip analyses showed that there were many similarities in genes with overexpression or low expression among the three patients (Fig. 4d). We used graphs to represent the functional enrichment of these genes (Fig. 4f), which confirmed the importance of EZH2 and HO-1 in progression.

### HO-1 regulates EZH2 via the pRB-E2F pathway

**Fig. 5:**
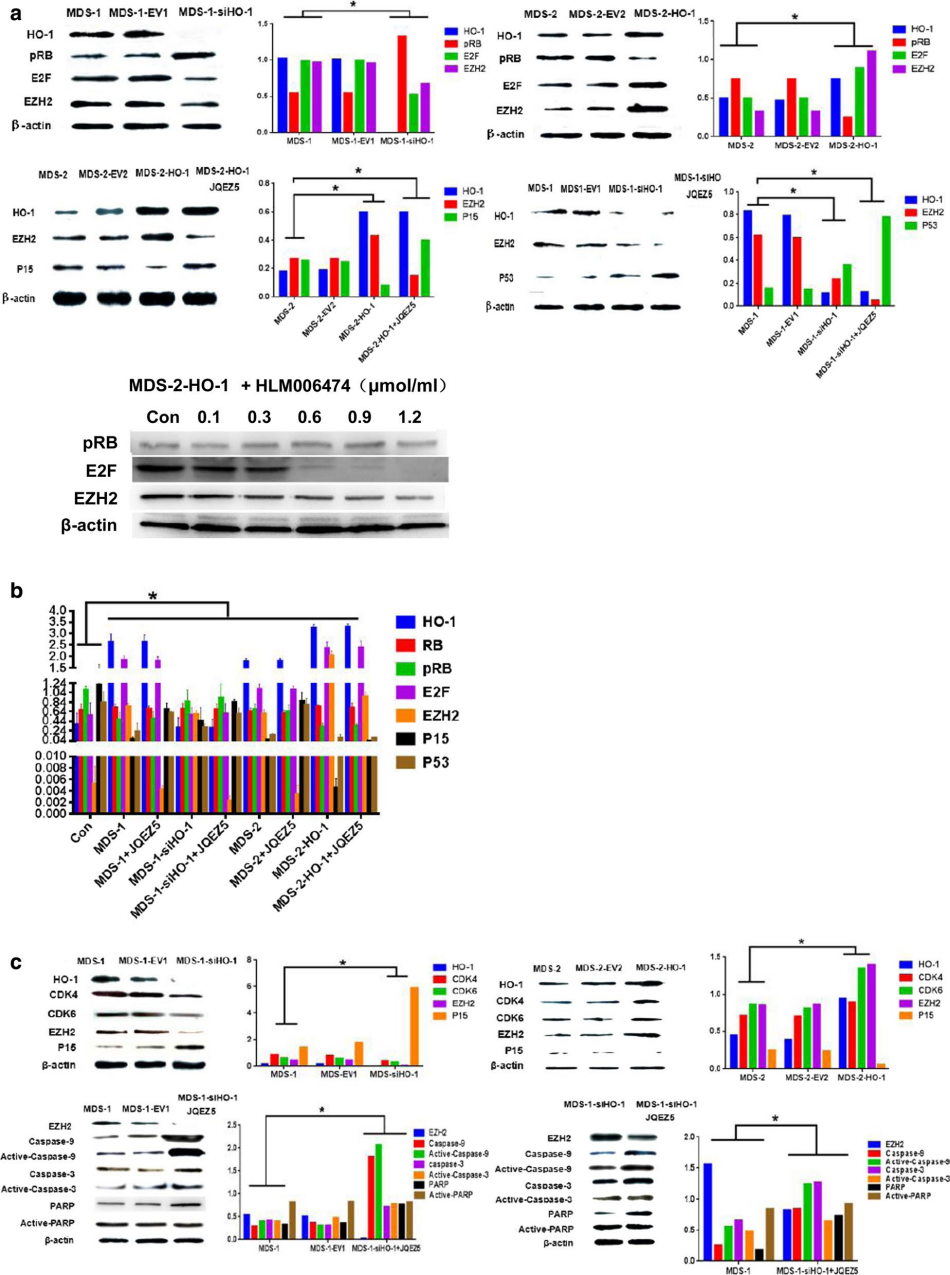

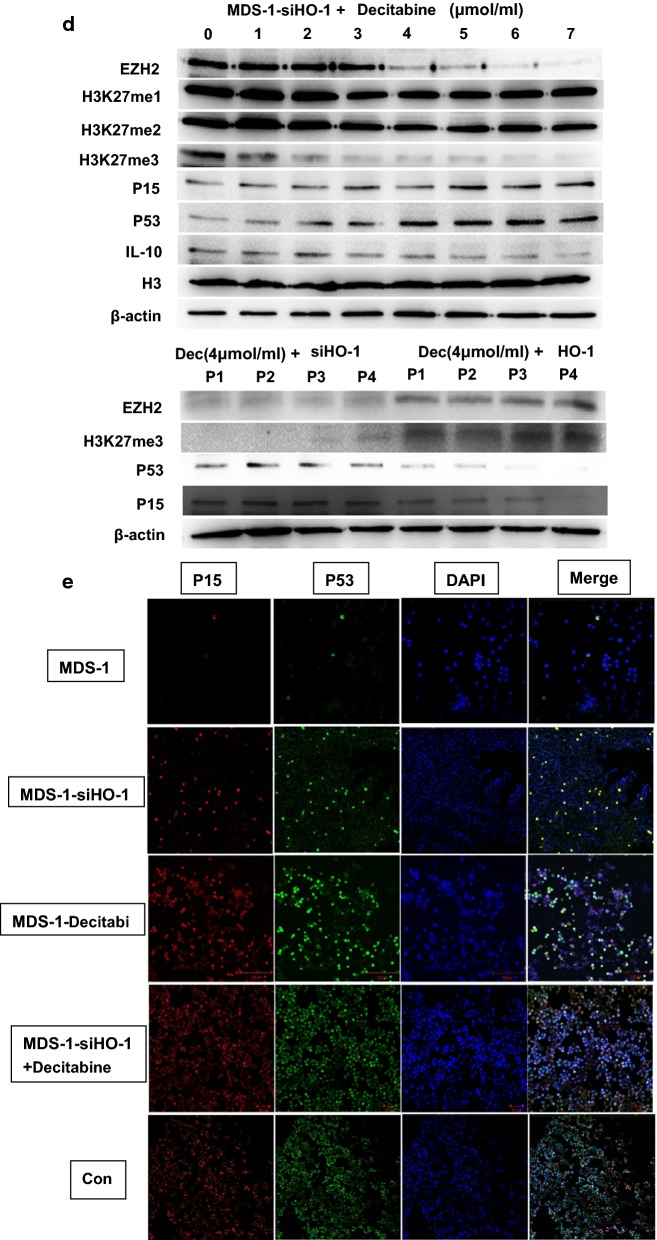
HO-1 regulates EZH2 via the pRB-E2F pathway. **a** HO-1 expression was regulated by a lentivirus (HO-1) or small interfering RNA (siHO-1). EV1 and EV2 represent the corresponding empty carriers. MDS1-si-HO-1 indicates downregulation of HO-1 expression. MDS2-HO-1 indicates upregulation of HO-1 expression. We divided these MDS-2-HO-1 cells into a control group (Con) and 5 experimental groups. The control group was not treated with HLM006474. We used different concentrations of HLM006474 to treat the experimental group cells. **b** After regulating HO-1, the expression levels of downstream genes were very different. We used three rulers to represent the value. **c** We regulated HO-1 and detected apoptotic genes (caspase-3, caspase-9 and PARP) and cycle-related genes (CDK4 and CDK6). **d** Dec represents decitabine. P1-P4 represents four MDS patients. These four MDS patients had overexpressed HO-1. They were resistant to decitabine and progressed to AML. **e** The expression of proteins in the cells was detected by laser scanning confocal microscopy. DAPI was used for nuclear staining. Merge refers to the overlap image. We also used the Quantity one software to analyze gray values. The relevant values are shown in the histogram. Each sample was normalized to related β-actin expression. All the experiments were repeated, *P < 0.05 (statistically significant)

We identified patient 3 as MDS1 and patient 7 as MDS2. HO-1 in MDS1 cells was silenced by siRNA (MDS-1-siHO-1). We also upregulated HO-1 expression in MDS2 cells with a lentivirus (MDS-2-HO-1). We divided these MDS-2-HO-1 cells into a control group (Con) and 5 experimental groups. The control group was not treated with HLM006474. We found that HO-1 could regulate EZH2 via the pRB-E2F pathway. Notebly, the EZH2 level in MDS-2-HO-1 cells did not increase when we inhibited E2F with the specific inhibitor HLM006474. The higher the concentration of HLM006474 was, the lower the EZH2 expression in MDS-2-HO-1 cells. We also used Quantity one software to analyze gray values, and the relevant values are shown in a histogram (Fig. 5a). Real-time PCR results also proved that HO-1 could regulate EZH2 via the pRB-E2F pathway (Fig. 5b). EZH2 is directly downstream of the pRB-E2F pathway and thus affects cell proliferation and apoptosis. We also used Quantity one software to analyze gray values, and the relevant values are shown in a histogram, (Fig. 5c). After HO-1 was inhibited, decitabine was able to enhance the expression of P15 and P53 in MDS cells. Moreover, when HO-1 was overexpressed in cells from four MDS patients (P1, P2, P3 and P4) that were then treated with decitabine, we found that HO-1 counteracted the anticancer effect of decitabine (Fig. 5d). We also used laser scanning confocal microscopy to examine previous Western blotting results. We used normal donor cells as control cells. Positive staining for P15 was more obvious in the control cells (Con) than in MDS-1, MDS-1-siHO-1 or MDS-1-Decitabi cells. Positive staining for P53 was also more obvious in the control cells (Con) than in MDS-1, MDS-1-siHO-1 or MDS-1-Decitabi cells. Inhibiting HO-1 significantly enhanced the ability of decitabine to induce P15 and P53 (Fig. 5e).

## Discussion

**Fig. 6:**
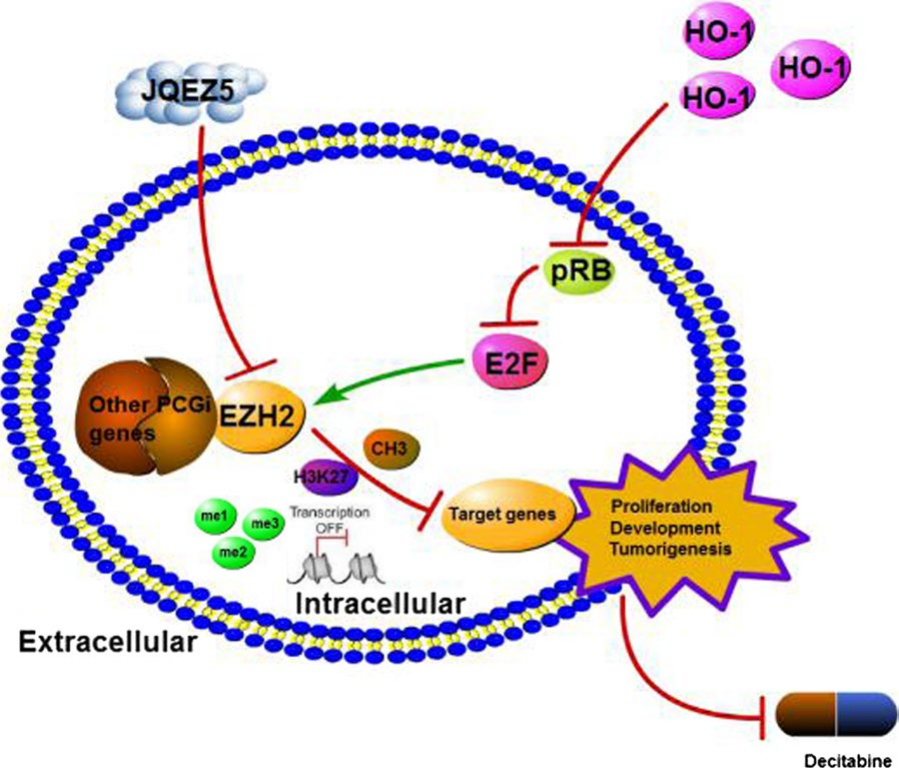
The mode of action of Ho-1 and EZH2 in MDS. A pictorial conclusion depicts the mode of action of HO-1 and EZH2 in MDS

In recent years, the number of MDS patients in our hospital has increased. Some high-risk or very high-risk patients progress to AML and quickly die while waiting to undergo stem cell transplantation [18]. Conventional treatment regimens do not relieve their symptoms. The prognosis of MDS is related to many factors that are very complex [19]. Some studies have suggested that decitabine may be effective against high-risk MDS [20, 21]. In our study, chemotherapy (decitabine or 5-azacytidine) stimulated MDS patients to produce large amounts of HO-1. Thus, patients had several risk factors, such as resistance to chemotherapeutic drugs, that might be due to HO-1 effects. In this study, we proved that HO-1 protected MDS cells by inhibiting apoptosis induced by JQEZ5 and decitabine. Previously, we were more concerned with the role of HO-1 in inflammation [22], but now we noticed that HO-1 could affect P15INK4B (P15) and TP53 (P53). P15 plays an important role in hematological tumors, and its expression is abnormal in approximately 50% of AML and MDS patients [23]. It has been reported that the kinase activity of P15 is suppressed by CDK4 and CDK6. Silencing P15 allows tumor cells to escape scrutiny at the cell cycle level [24], and P15 expression is upregulated when EZH2 expression is decreased [25, 26]. Based on our experimental results, HO-1 may negatively affect P15 in MDS. P53 is also associated with the prognosis of MDS patients and can be used to determine whether allogeneic hematopoietic stem cell transplantation (HSCT) would be successful in patients [27]. However, P53 is also a target of EZH2 and is inhibited when EZH2 is overexpressed [28]. We found that EZH2 was a downstream gene of HO-1; however, its role in MDS has not been fully elucidated. EZH2 is positively correlated with HO-1. EZH2 catalyzes the methylation of P53 and P15. A study showed that EZH2 downregulation in lung cancers could be used as a treatment [29]. Interestingly, a study suggested that the loss of EZH2 could contribute to leukemia transformation [30]. However, none of these studies discussed the association between HO-1 and EZH2 in MDS. HO-1 is able to induce EZH2 production. After excess EZH2 significantly inhibits P15 and P53, cells will continue to deteriorate. The effect of using decitabine as a monotherapy for the treatment of high-risk and very high-risk MDS patients is not satisfactory. We believe that inhibiting of HO-1 can help decitabine promote the expression of P53 and P15. We have included a pictorial conclusion depicting the mode of action of HO-1 and EZH2 in MDS (Fig. 6). High expression of P53 and P15 can inhibit tumor cell progression and promote apoptosis. A 2016 study by the Anderson Cancer Center indicated that low expression of EZH2 was an important prognostic marker in patients without chromosome 7 alteration [31]. Overexpression of EZH2 has also been associated with the diagnosis of MDS [32]. We believe that HO-1 is also related to the pathogenesis of MDS. Hence, it is reasonable to suggest that decitabine fails to treat some high-risk and very high-risk MDS patients who have high levels of EZH2 and HO-1.

Studies have shown that the pRB-E2F pathway is involved in the regulation of tumor cell function [33]. EZH2 is a gene downstream of the pRB-E2F pathway in MDS and is involved in the activation of oncogenes [34]. Inhibiting this pathway is therefore very important in cancer treatment [35, 36]. If high-risk or very high-risk MDS cells have an activated pRB-E2F pathway and EZH2 overexpression, the patients from whom the cells were isolated progress to AML. In our in vivo experiments, this phenotype also led to MDS cells infiltrating the mouse bone marrow in a short period of time. We suggest that these tumor cells destroyed the normal hematopoietic environment in the mice and consumed various growth factors. The pRB-E2F pathway can affect cell proliferation and apoptosis by regulating P53 and P15. A study found that P53 could also counteract pRB-E2F [37]. The balance between P53 and pRB-E2F is key to the normal growth of cells. HO-1 breaks the balance between them and makes it difficult for chemotherapeutic drugs to eliminate tumor cells.

Our experiments did not detail the characteristics of low-risk MDS patients because the expression of HO-1 and EZH2 in low-risk MDS is not significantly different from that in normal donors. Decitabine is not a preferred treatment for low-risk MDS patients, and the prognosis of low-risk MDS patients is generally better than that of high-risk MDS patients [38]. In total, 25% of patients with low-risk MDS progress to AML within 10.8 years [4]. Currently, the Food and Drug Administration (FDA) suggests that high-risk or very high-risk MDS patients be treated with decitabine. However, the results are unsatisfactory [39, 40]. For some high-risk or very high-risk MDS patients, EZH2 can be suppressed by demethylation drugs. Nevertheless, we found that if high HO-1 expression remained unchanged in patients, HO-1 functioned as an EZH2 catalyst. Thus, EZH2 expression in patients could be increased again when they were treated with demethylation drugs. In addition, HO-1 expression would abnormally increased after chemotherapy, which formed a cycle. Although JQEZ5 can significantly inhibit EZH2 expression in vitro, HO-1 expression in tumor cells still guarantees their survival. In summary, HO-1 regulated EZH2 expression via the pRB-E2F pathway, which was responsible for MDS progression to AML. High-throughput sequencing helped discover many pathways that may play a role in disease progression. However, we chose factors that were overlooked and then conducted in-depth research. If we do not pay attention to the mechanisms of drug resistance and overcome them, new drugs will not improve the prognosis of high-risk patients. Our experiments suggest that HO-1 is a potential target for the treatment of MDS. However, there is not enough clinical evidence to show that targeting HO-1 is absolutely safe in vivo. The collateral damage associated with targeting HO-1 is not clear. Many chemotherapeutic drugs inevitably have adverse effects on patients [41]. Our research aimed to provide a new perspective for MDS therapy. Increasingly people are paying attention to the impact of HO-1 on the treatment of leukemia, and thus, this molecule may be a target for preventing MDS progression to AML and the development of resistance.

## Conclusion

In conclusion, we demonstrated that EZH2 expression was associated with HO-1 expression in high-risk and very high-risk MDS patients. HO-1 could influence MDS drug resistance and progression into AML. The regulation of EZH2 might be mediated by the activation of the pRB-E2F pathway. Our study provides an important clue to the role of HO-1 to facilitate the development of EZH2-directed diagnostics and therapeutics for MDS.

## Data Availability

The datasets used and/or analyzed in this study will be made available by the authors on reasonable request.

## Abbreviations

MDS: myelodysplastic syndrome
AML: acute myeloid leukemia
EZH2: enhancer of zeste homologue 2
HO-1: Heme oxygenase-1
WPSS: WHO prognostic scoring system
FITC: fluorescein isothiocyanate
PI: propidium iodide
HSCT: hematopoietic stem cell transplantation
RA: refractory anemia
RCMD: refractory with multilineage dysplasia
RAEB: RA with excess of blasts
RAEB-t: RA with excess of blasts in transformation
IHC: immunohistochemistry
H&E staining: hematoxylin and eosin staining
EXP: experimental
Con: control
KEGG: Kyoto Encyclopedia of Genes and Genomes
siRNA: small interfering RNA
EV: empty plasmid vectors
L-HO-1: lentivirus encoding HO-1
si-HO-1: small interfering RNA specific for HO-1
WHO: World Health Organization
